# Does Gender Influence Lithium-Induced Nephrotoxicity?

**DOI:** 10.1192/j.eurpsy.2026.11192

**Published:** 2026-07-31

**Authors:** A. Yildiz, E. Sevim

**Affiliations:** Department of Biochemistry, Başakşehir Çam & Sakura City Hospital, İstanbul, Türkiye

## Abstract

**Introduction:**

Lithium remains the gold standard for bipolar disorder maintenance therapy, but its narrow therapeutic index and potential nephrotoxicity require close monitoring. Lithium-induced Chronic Kidney Disease (CKD) results from tubular and interstitial toxicity, leading to decreased estimated Glomerular Filtration Rate (eGFR) and elevated serum creatinine. Given lithium’s similarity to sodium and its renal tubular interactions, monitoring eGFR, creatinine, and sodium alongside serum lithium levels allows earlier detection of renal effects and better assessment of pharmacokinetic changes.

**Objectives:**

To evaluate the potential nephrotoxic effects of lithium by comparing renal biochemical parameters (sodium, creatinine, and eGFR) between lithium-treated patients and healthy controls in a one-year retrospective study.

**Methods:**

Lithium-treated patients and age- and sex-matched healthy controls were included. Sodium and creatinine were measured with the Roche Cobas 8000 analyzer, and eGFR was calculated using the CKD-EPI formula. Non-parametric data (p<0.05) were analyzed using the Mann–Whitney U test.

**Results:**

In females receiving lithium therapy, serum creatinine levels were significantly higher (p<0.05) and eGFR values were significantly lower (p<0.05) compared to the control group, while no significant difference was observed in sodium levels (p=0.34). In males, eGFR was significantly lower in the lithium-treated group (p<0.05), whereas creatinine and sodium levels showed no significant differences between the groups (p>0.05)

**Table 1.** Renal Parameters in Lithium-Treated vs. Control Groups Table 1. Long description.

	Lithium-treated male patients n = 186		Healthy male controls n = 149		
	Mean± SD	Median	Mean± SD	Median	P value
Sodium (mEq/L)	139.80 ± 2.67	140	140.34 ± 2.04	140	0.63
Creatinine (mg/dL)	0.83 ± 0.19	0.81	0.82 ± 0.06	0.83	0.64
eGFR (mL/min/1.73 m^2^)	104.23 ± 17.50	106	117.64 ± 6.55	119	**P<0.05**

	Lithium-treated female patients n = 139		Healthy female controls n=183		
	Mean± SD	Median	Mean± SD	Median	P value
Sodium(mEq/L)	139.68± 2.81	140	139.51± 2.36	139	0.34
Creatinine(mg/dL)	0.84± 0.14	0.80	0.66± 0.08	0.65	**P<0.05**
eGFR(mL/min/1.73 m^2^)	100.94± 15.56	102	113.68± 9.67	116	**P<0.05**

* p<0.05: statistically significant, SD: Standard Deviation, eGFR: estimated Glomerular Filtration Rate

**Conclusions:**

Lithium therapy reduces eGFR in both sexes, increasing the risk of renal dysfunction. The more pronounced increase in creatinine and decrease in eGFR observed in females suggest that the nephrotoxic effects of lithium may vary by sex. Therefore, in patients receiving lithium treatment, regular monitoring of renal parameters such as eGFR and creatinine, in addition to serum lithium levels, is clinically important to ensure patient safety and optimal therapeutic outcomes.

**Disclosure of Interest:**

None Declared

## Long descriptions

### Table 1. Long description

The first table displays data for males (186 lithium-treated, 149 controls).

* Sodium (mEq/L): Lithium mean 139.80 plus or minus 2.67, median 140; Control mean 140.34 plus or minus 2.04, median 140; P value 0.63.

* Creatinine (mg/dL): Lithium mean 0.83 plus or minus 0.19, median 0.81; Control mean 0.82 plus or minus 0.06, median 0.83; P value 0.64.

* e G F R (m L / min / 1.73 m super 2): Lithium mean 104.23 plus or minus 17.50, median 106; Control mean 117.64 plus or minus 6.55, median 119; P value less than 0.05.

The second table displays data for females (139 lithium-treated, 183 controls).

* Sodium (mEq/L): Lithium mean 139.68 plus or minus 2.81, median 140; Control mean 139.51 plus or minus 2.36, median 139; P value 0.34.

* Creatinine (mg/dL): Lithium mean 0.84 plus or minus 0.14, median 0.80; Control mean 0.66 plus or minus 0.08, median 0.65; P value less than 0.05.

* e G F R (m L / min / 1.73 m super 2): Lithium mean 100.94 plus or minus 15.56, median 102; Control mean 113.68 plus or minus 9.67, median 116; P value less than 0.05.

Navigate back to Table 1..

